# Job satisfaction, burnout, and turnover intention among primary care providers in rural China: results from structural equation modeling

**DOI:** 10.1186/s12875-020-1083-8

**Published:** 2020-01-15

**Authors:** Haipeng Wang, Yinzi Jin, Dan Wang, Shichao Zhao, Xingang Sang, Beibei Yuan

**Affiliations:** 1 School of Health Care Management, Shandong University, Jinan, 250012 China; 2 NHC Key Laboratory of Health Economics and Policy Research (Shandong University), Jinan, 250012 China; 3 Department of Global Health, School of Public Health, Peking University, Beijing, 100191 China; 4 China Center for Health Development Studies, Peking University, Beijing, 100191 China; 5 School of Public Administration, Shandong Normal University, Jinan, 250014 China; 6 Health Commission of Weifang, Weifang, 261061 Shandong China

**Keywords:** Job satisfaction, Burnout, Turnover intention, Primary care providers, Structural equation modeling

## Abstract

**Background:**

Low job satisfaction, severe burnout and high turnover intention are found to be prevalent among the primary care providers (PCPs) in township health centers (THCs), but their associations have received scant attention in the literature. In light of this, this study aims to examine the relationships between job satisfaction, burnout and turnover intention, and explore the predictors of turnover intention with a view to retaining PCPs in rural China.

**Methods:**

Using the multistage cluster sampling method, a cross-sectional survey was conducted in Shandong Province, China. 1148 PCPs from 47 THCs participated in this study. Job satisfaction, burnout and turnover intention were measured with a multifaceted instrument developed based on the existing literature, the Maslach Burnout Inventory and the participants’ responses to a Likert item drawn from the literature, respectively. The relationships of the three factors were examined using Pearson correlation and structural equation modeling, while the predictors of turnover intention were investigated using multivariate logistic regression.

**Results:**

The subscale that the PCPs were most dissatisfied with was job rewards (95.12%), followed by working environment (49.65%) and organizational management (47.98%). The percentages of the PCPs reporting high-levels of emotional exhaustion, depersonalization and reduced personal accomplishment were 27.66, 6.06, and 38.74%, respectively. About 14.06% of the respondents had high turnover intention. There was a significant direct effect of job satisfaction on burnout (γ = − 0.52) and turnover intention (γ = − 0.29), a significant direct effect of burnout on turnover intention (γ = 0.28), and a significant indirect effect (γ = − 0.14) of job satisfaction on turnover intention through burnout as a mediator. Work environment satisfaction, medical practicing environment satisfaction, and organizational management satisfaction proved to be negative predictors of turnover intention (*p* < 0.05), whereas reduced personal accomplishment was identified as a positive predictor (*p* < 0.05).

**Conclusions:**

Plagued by low job satisfaction and severe burnout, the PCPs in rural China may have high turnover intentions. Job satisfaction had not only negative direct effects on burnout and turnover intention, but also an indirect effect on turnover intention through burnout as a mediator. Targeted strategies should be taken to motivate and retain the PCPs.

## Background

As the hinge of three-tier health-care network in rural China, township health centers (THCs) are responsible for basic medical care and public health service delivery for rural populations [1–3]. As direct providers of health services, primary care providers (PCPs) constitute a key element of the healthcare system [4, 5], and the availability of enough skilled and motivated PCPs is critical to delivering effective health services and improving health outcomes [6]. However, PCPs in rural areas across the globe are confronted with similar challenges which can negatively impact the delivery of healthcare, particularly in low and middle-income countries [7]. It is no exaggeration to say that for all countries worldwide, motivating and retaining the health workforce in rural and remote areas is central to their health system strengthening initiatives. Health policy makers, however, have for a long time focused primarily on increasing human resources supply and quality, paying insufficient attention to the job satisfaction, burnout, and turnover intention of the PCPs in THCs.

In China, the rural PCPs in THCs include doctors, nurses, public health providers (PHPs) employed by THCs [8]. The rural PCPs are often inadequately trained, and about half of them fail to meet the educational requirement for a licensed assistant doctor (i.e. junior medical college) [9]. Continuing education for the PCPs is also insufficient. On the contrary, almost all hospital-based providers are licensed doctors. In addition to the low education background and inadequate capacity, rural PCPs faced challenges in financial renumeration which have been found to be the most demotivating factor for them [10]. Despite their pay rise, the salary gap has widened over the past decade between health professionals working in primary healthcare facilities (¥57,000 in 2017) and public hospitals (¥131,000 in 2017) [11].

Job satisfaction, a major determinant of the motivation and performance of health workers [8], is generally low among PCPs in China [9]. According to the 2011 China primary care workforce survey, the overall rate of job satisfaction was only 47.6% [12]. Job satisfaction is defined as the extent that the health workers are positive, negative or affective towards their work. It depends not only on the nature of the job, but also the expectation health workers have of what their job should provide [7, 13, 14]. Job satisfaction can be measured globally as the general satisfaction with a job, or in a multi-faceted way based on the satisfaction with the specific dimensions such as salary, benefits, career development, training, work relationships, promotion, management, work environment, recognition and supervision [3, 14]. Job satisfaction of health workers could be influenced by a range of individual and organizational factors including personality, work itself, work organization, remuneration, workloads, administration, and leadership styles [1, 7, 15]. Low job satisfaction might lead to work stress, occupational burnout and turnover intention [3, 7].

High occupational burnout is also widespread among PCPs in China [9]. A systematic review showed that the overall burnout rate of doctors in China lay between 66.5 and 76.9%, with the high burnout rate ranging from 12.1 to 25.4% [16]. Prolonged stresses, if not effectively handled, can result in burnout, a negative psychological syndrome involving physical, emotional and mental states and responses [17–19]. According to Maslach, burnout is a reaction to the imbalance between work-related demands and personal resources and it is manifested through the three dimensions of emotional exhaustion, depersonalization and reduced personal accomplishment [5, 20]. A national survey conducted among 10,626 primary healthcare doctors revealed that 41% of the respondents felt highly exhausted, 37% highly depersonalized, and 34% reported a low sense of personal accomplishment [21]. Previous studies have identified excessive workload, practice setting, working condition, lack of support, and role ambiguity as specific factors related to burnout symptoms, which in turn would lead to turnover intention [22].

Turnover intention is another prevalent challenge among PCPs in China. It is reported that the proportion of PCPs with clear turnover intentions ranged from 13.1 to 58.0% [23–25]. Turnover can be defined as the process whereby employees leave an organization for other jobs [5]. Deemed as a major predictor of turnover behavior, turnover intention is used to reveal the employees’ expressed intention of leaving their current job within a certain period [7], and it is associated with various factors including work role and stress, workplace violence, work environment and experience, organizational system and climate, job satisfaction and burnout [18]. Given the heavy workloads, unsustainable work environments, poor living conditions and infrastructure that the health workers are facing with, it would be a big challenge to retain them in rural areas [3]. High turnover intention, resulting in a heavier workload, undermined team morale and lower work efficiency, would subsequently hinder working performance [26]. Moreover, high turnover rate is a major contributor to the current PCP shortages in rural areas.

Among the large body of literature on the health workers’ job satisfaction, burnout and turnover intention, some studies have established their correlation with one another and their repercussions for the health workforce [5, 22, 26]. It was confirmed that low job satisfaction and its dimensions can significantly contribute to occupational burnout of healthcare staff, and those experiencing high levels of burnout are more likely to have turnover intention and leave their positions [17, 22]. In China, the relationships between job satisfaction, burnout and turnover intention have been studied only on physicians in urban medical institutions and nurses in tertiary hospitals [17, 18], whereas little is known about their correlation and relevance to the rural PCPs. Furthermore, THCs have experienced substantial change since the introduction of New Health System Reform (NHSR), which has had significant impact on the function of primary care delivery in these settings. Therefore, we conducted this study to examine the interrelationship between job satisfaction, burnout and turnover intention, and explore the predictors of turnover intention among the PCPs in rural China.

## Methods

### Study design and sample

This is a cross-sectional study conducted in Shandong province in December 2017. Shandong is a major province in east China with a vast area and large population. It is named as “small China” because of the same geographical distribution of different economically developed regions: eastern, middle and western regions representing the less-, middle-, and high-developed status. In Shandong, we used a multistage random cluster sampling to select THCs and PCPs. At first, three counties (Shouguang, Huantai and Yanggu) were selected based on an equal representation of high-, middle- and low-level economic region according to the gross domestic product per capita. Then all the THCs in the three counties were selected, including 16 from Shouguang, 13 from Huantai, and 18 from Yanggu. All the PCPs in the selected THCs were the sample in this study.

### Data collection

The research team paid a visit to the selected THCs to collect data. During the visit we invited all the PCPs on duty to participate in the survey. Each participant completed a self-administered questionnaire independently, with research staffs available to address their questions. About 82% of eligible PCPs participated in the survey. The final participants consisted of 1148 PCPs including 699 doctors, 136 nurses and 313 PHPs.

This study was approved by the Ethics Committee of Peking University Health Science Center (code of ethics: PKU201412128). All participants were voluntary and their informed consents for participation in the survey were obtained prior to the questionnaire administration.

### Measures and variables

We assessed job satisfaction with a multifaceted instrument which was developed based on the existing literature and included intrinsic, extrinsic, occupational and organizational factors [17]. Final measures consisted of five subscales including job-itself satisfaction with 4 items (job designing, workload, autonomy, training), work environment satisfaction with 3 items (work environment, drug need, equipment need), job rewards satisfaction with 3 items (wage, assessing method of wage, benefit), medical practicing environment satisfaction with 3 items (peer relationship, doctor-patient relationship, and supporting among departments), and organizational management satisfaction with 5 items (title promotion, position promotion, performance feedback, rewards and punishments, participation in decision making). We asked the participants to rate their satisfaction according to how each item contributed to their work satisfaction. Their responses were measured on a 5-point Likert scale from 1 (very dissatisfied) to 5 (very satisfied), To evaluate the status of job satisfaction, the 5-point Likert scale was collapsed into a binary category of dissatisfied (very dissatisfied, dissatisfied, moderate) and satisfied (satisfied, very satisfied). For the five subscales, their mean scores were computed and their Cronbach’s alphas were 0.816, 0.856, 0.760, 0.748, and 0.886, respectively, indicating high levels of reliabilities.

We measured occupational burnout using the 22-item Maslach Burnout Inventory (BMI) which includes emotional exhaustion (9 items), depersonalization (5 items), and reduced personal accomplishment (8 items) [20, 27]. The PCPs’ feelings were elicited on a 7-point Likert scale: never having those feelings (0), having those feelings a few times a year (1), having those feelings one time a month (2), having those feelings a few times a month (3), having those feelings one time a week (4), having those feelings a few times a week (5), and having those feelings everyday (6). The Cronbach’s alphas for the three subscales were 0.873, 0.734, and 0.859, respectively, considered acceptable in reliability test. For the three subscales, the mean scores were calculated and the total scores were collapsed into a binary category to evaluate the status of burnout. The cutoff scores were set at more than 27 and 13 for emotional exhaustion and depersonalization respectively, and no more than 31 for personal accomplishment [28].

Turnover intention was measured by the participants’ responses to the Likert item: “Do you have the thoughts of leaving this faculty for other jobs elsewhere at present?”, with their responses being rated from 1 (highly disagree) to 5 (highly agree). Then the 5-point Likert scale was collapsed into a binary category of disagree (highly disagree, disagree, average) and agree (agree, highly agree) to evaluate the status of turnover intention.

We also collected a number of covariates regarding the rural PCPs. Social-demographic variables included age (< 30, 30–39, 40–49, ≥50), gender (female, male), marriage status (married, single/divorced/widowed), education background (high school or below, junior college, bachelor and above), and monthly income in CNY (< 3000, 3000–4999,≥5000). Work-related variables included type of PCPs (physician, nurse, PHP), technical title (senior/vice-senior, intermediate, primary, no title), employment mode (formal, casual), years of working (< 5, 5–9, ≥10), and hours worked per week (≤40, 41–48, 49–56, ≥57).

### Statistical analysis

First, we obtained frequency (N) and percentage (%) statistics to show the socio-demographic and work-related characteristics of the participants, and conducted Chi-square tests to determine the differences among three types of PCPs. Second, we calculated scores (Mean ± SD) and binary category variables (N, %) of job satisfaction, burnout and turnover intention, and conducted Chi-square tests to determine the differences among three types of PCPs. Third, we calculated the Pearson correlation coefficients to determine the associations among job satisfaction, burnout and turnover intention. Next, we employed structural equation modeling (SEM) to verify the path and synthetic relationship among job satisfaction, burnout, and turnover intention. Maximum likelihood estimation (MLE) was performed to estimate these parameters in SEM. Lastly, we applied multivariate logistic regression to examine the factors of turnover intention, where job satisfaction and burnout syndrome were taken as principal predictors and covariates were used as controlled variables. Odds ratios (ORs) with corresponding 95% confidence intervals (CI) were reported. All statistical analyses were performed using Stata 15.1 and AMOS 7.0. Statistical significance was set at *P* < 0.1 and *P* < 0.05.

## Results

### Participants’ characteristic

Out of 1148 PCPs, the majority were female (64.72%). The minority were under the age of 30 (16.23%) or over the age of 50 (7.46%). Most of the PCPs were living with a partner (90.66%). More than 60% of the PCPs received low levels of education. Nearly 27% of the PCPs earned less than 3000 CNY per month. Physicians accounted for 60.89% of the PCPs surveyed, followed by PHPs (27.26%) and nurses (11.85%). Only 1.58% of the PCPs had senior or vice-senior technical titles, whereas 19.28% of the PCPs had no technical titles. Most of the PCPs were practicing more than 40 h per week (92.92%), under formal employment contract (76.11%). About a fifth (21.17%) of the PCPs had worked for less than 5 years, while nearly 18% had 5–9 years of service. There were significant differences in the majority of these characteristics among three types of the PCPs (*P* < 0.05). (Table 1).

**Table 1:** Socio-demographic and work-related characteristics of participants (N, %)

	Overall (1148)	Physicians (699)	Nurses (136)	PHPs (313)	*P* value
Gender					
Male	404 (35.28)	279 (40.09)	14 (10.29)	111 (35.46)	< 0.001
Female	741 (64.72)	417 (59.91)	122 (89.71)	202 (64.54)	
Age in years					
< 30	185 (16.23)	107 (15.42)	22 (16.30)	56 (18.01)	0.483
30–39	472 (41.40)	288 (41.50)	65 (48.15)	119 (38.26)	
40–49	398 (34.91)	249 (35.88)	40 (29.63)	109 (35.05)	
≥ 50	85 (7.46)	50 (7.20)	8 (5.93)	27 (8.68)	
Marital status					
Unmarried	107 (9.34)	67 (9.61)	13 (9.56)	27 (8.63)	0.879
Married	1039 (90.66)	630 (90.39)	123 (90.44)	286 (91.37)	
Education					
High school or below	228 (19.87)	110 (15.74)	24 (17.78)	94 (30.03)	< 0.001
Junior college	481 (41.94)	272 (38.91)	68 (50.37)	141 (45.05)	
Bachelor and above	438 (38.19)	317 (45.35)	43 (31.85)	78 (24.92)	
Monthly income (CNY)					
< 3000	285 (26.56)	134 (20.62)	25 (19.69)	126 (42.57)	< 0.001
3000–4999	437 (40.73)	273 (42.00)	52 (40.94)	112 (37.84)	
≥ 5000	351 (32.71)	243 (37.38)	50 (39.37)	58 (19.59)	
Technical title					
Senior/Vice-senior	18 (1.58)	16 (2.30)	2 (1.49)	0 (0.00)	< 0.001
Intermediate	398 (34.88)	274 (39.37)	47 (35.07)	77 (24.76)	
Primary	505 (44.26)	317 (45.55)	59 (44.03)	129 (41.48)	
No title	220 (19.28)	89 (12.79)	26 (19.40)	105 (33.76)	
Employment mode					
Formal	873 (76.11)	559 (79.97)	93 (68.89)	221 (70.61)	0.001
Casual	274 (23.89)	140 (20.03)	42 (31.11)	92 (29.39)	
Years of working					
< 5	238 (21.17)	120 (17.47)	29 (22.31)	89 (28.99)	0.001
5–9	200 (17.79)	134 (19.51)	17 (13.08)	49 (15.96)	
≥ 10	686 (61.03)	433 (63.03)	84 (64.62)	169 (55.05)	
Working hours per week					
≤ 40	78 (7.08)	56 (8.35)	7 (5.26)	15 (5.05)	< 0.001
41–48	736 (66.85)	414 (61.70)	106 (79.70)	216 (72.13)	
49–56	192 (17.44)	117 (17.44)	16 (12.03)	59 (19.87)	
≥ 57	95 (8.63)	84 (12.52)	4 (3.01)	7 (2.36)	

Note: *PHPs* public health providers, *CNY* Chinese Yuan

### Job satisfaction, burnout and turnover intention

The PCPs were least satisfied with job rewards and most satisfied with medical practicing environment, with the mean scores ranging from 2.93 to 3.81. Among the job satisfaction subscales which were recorded in a binary category, medical practicing environment had the lowest dissatisfaction rate of 29.77%, while job rewards had the highest dissatisfaction rate of 95.12%. In terms of burnout, the mean scores of emotional exhaustion, depersonalization, and reduced personal accomplishment were 2.40, 0.93 and 4.15, respectively. As for the burnout subscales whose total scores were also collapsed into a binary category, the proportion reporting high emotional exhaustion, high depersonalization, and low personal accomplishment were 27.66, 6.06%, and 38.74, respectively. The observed mean score of turnover intention was 2.25 among the sampled PCPs, and 14.06% of them had high turnover intention. There were significant differences in job satisfaction and turnover intention (*P* < 0.05), but no significant differences (*P* > 0.05) in emotional exhaustion among three types of the PCPs. (Table 2).

**Table 2:** Job satisfaction, burnout and turnover intention among the PCPs in rural China

	Scores (Mean ± SD)	Dissatisfied/Severe/Intended (N, %)				
		Overall	Physicians	Nurses	PHPs	*P* value
Job satisfaction						
Job-itself satisfaction	3.76 ± 0.68	425 (38.92)	269 (40.33)	56 (43.41)	100 (33.78)	0.085
Working environment satisfaction	3.53 ± 0.75	564 (49.65)	384 (55.25)	69 (51.11)	111 (36.27)	< 0.001
Medical practicing environment satisfaction	3.81 ± 0.64	340 (29.77)	232 (33.43)	48 (35.56)	60 (19.17)	< 0.001
Job rewards satisfaction	2.93 ± 0.38	1052 (95.12)	653 (96.88)	128 (96.97)	271 (90.33)	< 0.001
Organizational management satisfaction	3.45 ± 0.77	522 (47.98)	344 (51.57)	60 (47.24)	118 (40.14)	0.005
Burnout (MBI)						
Emotional exhaustion	2.40 ± 1.19	301 (27.66)	186 (27.97)	36 (27.91)	79 (26.87)	0.938
Depersonalization	0.93 ± 1.01	67 (6.06)	39 (5.76)	12 (9.30)	16 (5.35)	0.253
Reduced personal accomplishment	4.15 ± 1.37	401 (38.74)	245 (38.46)	44 (37.61)	112 (39.86)	0.890
Turnover intention	2.25 ± 1.08	160 (14.06)	115 (16.64)	16 (11.85)	29 (9.29)	0.006

Note: *PHPs*, public health providers, *MBI* Maslach Burnout Inventory

### Pearson correlations between job satisfaction, burnout and turnover intention

Table 3 shows the correlations between the job satisfaction, burnout, and turnover intention of the PCPs. Turnover intention was significantly negatively correlated with each subscale of job satisfaction (*P* < 0.001), and positively associated with each subscale of burnout syndrome (*P* < 0.001). Out of the five subscales of job satisfaction, turnover intention had the strongest association with organizational management (*r* = − 0.401) and the weakest association with job rewards (*r* = − 0.294). As for the associations between turnover intention and the three subscales of burnout syndrome, the value of the correlation coefficient was 0.319 for emotional exhaustion, 0.225 for depersonalization, and 0.270 for reduced personal accomplishment.

**Table 3:** Correlation between job satisfaction, occupational burnout, and turnover intention

	Subscales	Pearson correlation coefficient with turnover intention	*P* value
Job satisfaction	Job-itself satisfaction	−0.377	< 0.001
	Working environment satisfaction	−0.342	< 0.001
	Medical practicing environment satisfaction	−0.378	< 0.001
	Job rewards satisfaction	−0.294	< 0.001
	Organizational management satisfaction	−0.401	< 0.001
Burnout (MBI)	Emotional exhaustion	0.319	< 0.001
	Depersonalization	0.225	< 0.001
	Reduced personal accomplishment	0.270	< 0.001

Note: *MBI* Maslach Burnout Inventory

### Path relationship between job satisfaction, burnout, and turnover intention

**Fig. 1:**
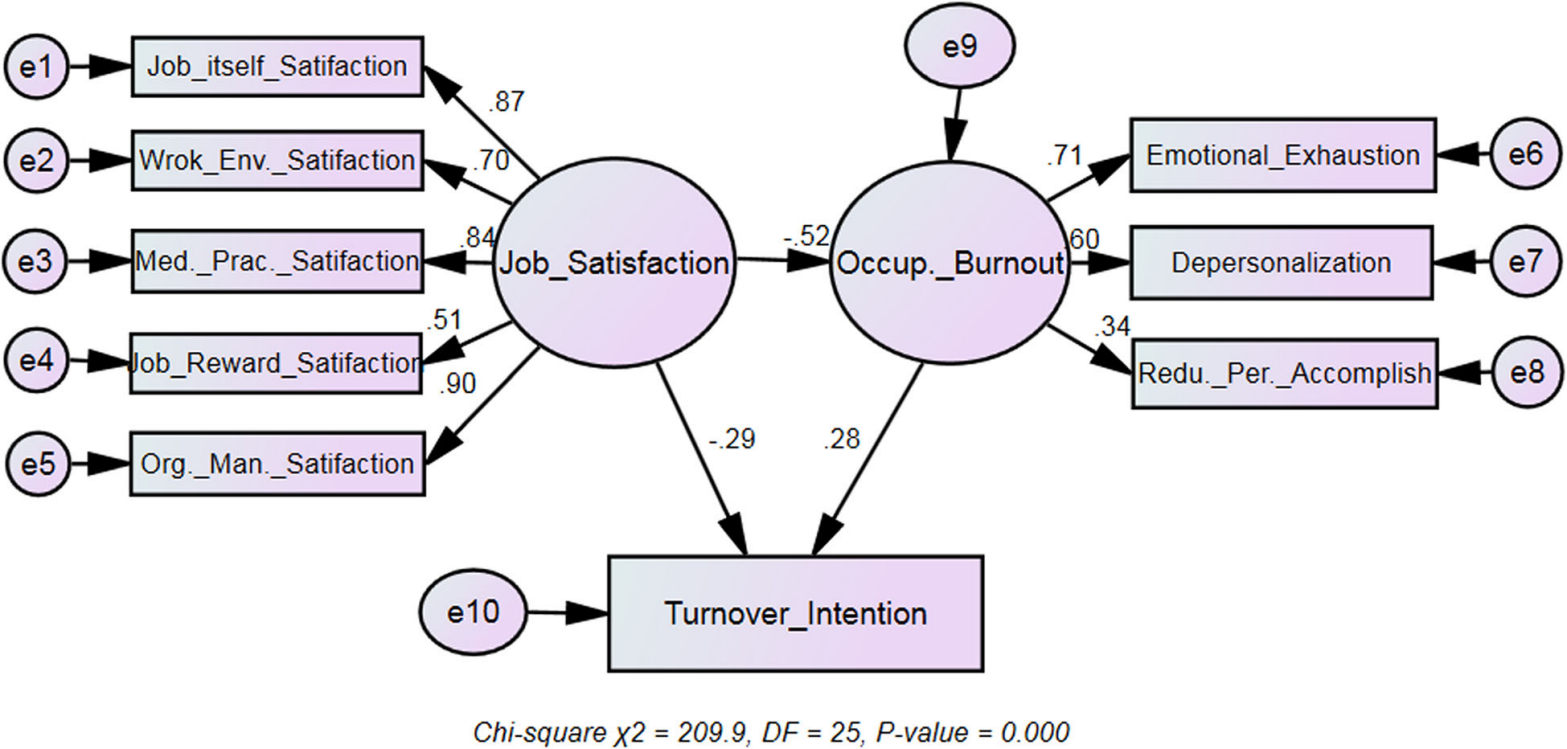
The path relationships between job satisfaction, occupational burnout and turnover intention examined using SEM

The model fit indices of the SEM were all within specifications (GFI = 0.962, NFI = 0.950, CFI = 0.956, IFI = 0.956, TLI = 0.936, RMSEA = 0.080, RMR = 0.050, and AGFI = 0.931), indicating good model fit. The edges between the dimensions in Fig. 1 represent direct relationships. The indirect effect of one dimension on another is equal to the product of the regression coefficients of the two directly connected dimensions. For job satisfaction, the standardized factor loadings ranged from 0.51 (job rewards satisfaction) to 0.90 (organizational management satisfaction), and for occupational burnout, the values ranged from 0.34 (reduced personal accomplishment) to 0.71 (emotional exhaustion). There was a direct effect of job satisfaction on occupational burnout (γ = − 0.52) and turnover intention (γ = − 0.29), a direct effect of occupational burnout on turnover intention (γ = 0.28), and also an indirect effect of job satisfaction on turnover intention through burnout as a mediator. For turnover intention, job satisfaction had smaller indirect effect (γ = − 0.52 × 0.28 = − 0.14) than its direct effect. The total effect of job satisfaction on turnover intention was − 0.43, equaling its direct effect (γ = − 0.29) plus indirect effect (γ = − 0.14).

### Multiple logistic regression analysis of the factors related to turnover intention

Male PCPs were 1.91 times more likely to have turnover intentions than female PCPs (*p* = 0.014) and the formal PCPs 2.01 times more likely than the casual PCPs (*p* = 0.097). In addition, the PCPs with 5–9 years of service have higher turnover intention than their counterparts with less than 5 years of service (OR = 2.46, p = 0.057), and the PCPs working more than 56 h per week have higher turnover intention than those working no more than 40 h per week (OR = 2.49, *p* = 0.098). In terms of the subscales of job satisfaction, work environment, medical practicing environment and organizational management proved to be negative predictors of turnover intention under the control of other variables (*p* < 0.05), whereas job itself and job rewards were not significantly related to turnover intention (*p* > 0.1). Among the subscales of burnout syndrome, reduced personal accomplishment was identified as a positive predictor of turnover intention (*p* < 0.05) (Table 4).

**Table 4:** Determinants of turnover intention by logistic regression analysis

Explanatory Variables	Categories	OR (95%CI)	*P* value
Socio-demographic variables			
Gender (ref. = Female)	Male	1.91(1.14, 3.18)	**0.014**
Age (ref. = < 30)	30–39	1.14(0.43, 3.00)	0.795
	40–49	1.22(0.40, 3.71)	0.721
	≥50	0.65(0.13, 3.36)	0.608
Marital status (ref. = Unmarried)	Married	1.18(0.42, 3.35)	0.757
Education (ref. = High school or below)	Junior college	1.21(0.54, 2.71)	0.647
	Bachelor and above	1.39(0.61, 3.17)	0.439
Monthly income (ref. = < 3000)	3000–4999	0.82(0.38, 1.75)	0.610
	≥5000	1.14(0.48, 2.69)	0.764
Work-related characteristics			
Provider type (ref. = Physician)	Nurse	0.72(0.32, 1.64)	0.434
	PHP	0.75(0.40, 1.41)	0.366
Technical title (ref. = Senior)	Intermediate	0.45(0.12, 1.72)	0.243
	Primary	0.48(0.12, 1.97)	0.308
	No title	0.38(0.07, 2.04)	0.262
Employment mode (ref. = Formal)	Casual	2.01(0.88, 4.62)	**0.097**
Years of service (ref. = < 5)	5–9	2.46(0.97, 6.20)	**0.057**
	≥10	1.52(0.58, 3.98)	0.390
Working hours per week (ref. = ≤40)	41–48	1.50(0.54, 4.20)	0.437
	49–56	1.14(0.36, 3.68)	0.819
	≥57	2.49(0.78, 7.92)	**0.098**
Job satisfaction subscales			
Job-itself satisfaction (ref. = Satisfied)	Dissatisfied	0.83(0.44, 1.59)	0.582
Working environment satisfaction (ref. = Satisfied)	Dissatisfied	2.55(1.32, 4.93)	**0.005**
Medical practicing environment satisfaction (ref. = Satisfied)	Dissatisfied	1.81(1.03, 3.18)	**0.041**
Job rewards satisfaction (ref. = Satisfied)	Dissatisfied	0.86(0.23, 3.20)	0.820
Organizational management satisfaction (ref. = Satisfied)	Dissatisfied	2.29(1.13, 4.62)	**0.021**
Burnout syndrome subscales			
Emotional exhaustion (ref. = Low-level)	High-level	1.33(0.80, 2.20)	0.270
Depersonalization (ref. = Low-level)	High-level	1.24(0.48, 3.23)	0.652
Reduced personal accomplishment (ref. = Low-level)	High-level	1.66(1.03, 2.67)	**0.036**
Constants		0.02(0.01, 0.20)	**0.002**

Note: *PHP* public health provider

## Discussion

This study presents critical information on the current profile of the job satisfaction, burnout, turnover intention and their relationships among the PCPs in rural China. As a whole, the PCPs were found to be generally dissatisfied with most dimensions of their work and working experience, especially job rewards and organizational management. A considerable proportion of PCPs were suffering from severe burnout syndrome, reduced personal accomplishment and emotional exhaustion in particular. Additionally, some PCPs reported high turnover intention. Job satisfaction had a significant negative direct effect on occupational burnout and turnover intention, and occupational burnout had a significant positive direct effect on turnover intention. Moreover, there was a significant indirect effect of job satisfaction on turnover intention through occupational burnout as a mediator. We should pay more attention to these observations, as they could not only lead to the brain drain, but also negatively impact the quality of primary health care and overall efficiency of health system in the long term [24, 29].

This study found that the PCPs were most dissatisfied with job rewards, with the satisfaction rate being only less than 5%. This finding is consistent with previous studies according to which PCPs were the least satisfied with income and benefits [1, 3, 12]. It is indicated that the PCPs have not enjoyed significant increase in income although the THCs received more funds from both central and local governments since the introduction of NHSR. Now in most areas, it is the government that is responsible for the PCPs’ salaries, which may not go up since higher revenues from clinical services and drug prescription are controlled and the government has also set a limit on the performance salary allocated from the net revenue of THCs. What’s more, THCs have witnessed a surge in their workloads since the introduction of the basic public health services program [23]. The heavy workload, coupled with stagnant income, is the major culprit for the general dissatisfaction with financial rewards and the dissatisfaction was exacerbated by perceived inequity [30]. Only when a worker feels rewarded fairly can he concentrate on the job instead of worrying about rewards [31]. These findings are echoed in similar studies which also indicated that the mismatch between income and workloads is a major contributor toward job dissatisfaction among PCPs [12]. Therefore, policymakers should explore initiatives to strike a balance between the PCPs’ job rewards and workload.

Furthermore, it is worth noting that the PCPs were very dissatisfied with the organizational management system within their THCs, which could be attributed to the great changes in the PCPs’ working conditions and supervision environments as a result of governments’ reform in primary health system in the last decade [32]. In addition, with their salaries gradually increased, PCPs will pay more attention to the organizational management than before. However, the organizational management system and internal operation mechanism have not concurrently been adapted and adjusted within THCs [9, 33], which are still troubled by many issues concerning career development and management environment such as an unreasonable standard of professional title promotion and a lack of professional managers. For example, the performance assessment measures applied by the basic public health services program are over strict and frequent, and have thus made PCPs in THCs feel not trusted. Previous studies have demonstrated that organizational factors affected the job satisfaction of employees [34], whilst the employees who received more formal and informal support exhibited higher work satisfaction [35]. Poor remuneration mechanisms and unsuitable professional promotion system were also the common factors for job dissatisfaction among urban PCPs [30]. These findings implied that managers in THCs should take measures to improve the internal organizational management, such as establishing incentive mechanisms and conducting performance assessments.

This study also revealed that a considerable proportion of PCPs were suffering from severe burnout syndrome. Compared with physicians in urban public hospitals in China [17], a lower proportion of the rural PCPs reported (28% vs 67%) high-levels of emotional exhaustion, but a higher proportion (39% vs 6%) reported high-levels of reduced personal accomplishment. The main explanation is that the rural PCPs are usually exposed to low job rewards and limited opportunities of career development, so the inability to fulfill their personal value has raised a great concern for them [1]. Therefore, health managers should introduce more incentives so that the rural PCPs can gain more recognition [36]. Similarly, according to a study conducted among French PCPs, 16.0% of the respondents reported emotional exhaustion, 33.8% depersonalization, and 38.9% low personal accomplishment [37]. It has been observed that factors influencing burnout include low reward, role overload, low decision authority, low support, and low organizational justice [16]. Moreover, since the basic public health services is mandated by government, PCPs have served as the providers of both basic public health services and basic medical care, thus being subject to much heavier workloads [25]. As for the public health services, there are explicit guidelines on work procedure to follow and performance targets to meet, hence the PCPs have to devote huge amounts of time to paperwork.

The stability of skilled PCPs is crucial for meeting the basic health service demands of residents. However, policy makers and organization managers pay more attention to cultivating PCPs than to the factors resulting in turnover intention [23]. This study found that more than 14% of the PCPs in THCs reported high turnover intention. As turnover intention has a significant positive correlation with turnover behavior, it is vital to further explore its current factors to avoid future brain drain. Generally, the PCPs’ intention to resign depends on to what extent their work demand is met [23]. We also found that turnover intention was negatively associated with job satisfaction and positively related to occupational burnout. When the PCPs experienced lower job satisfaction and higher occupational burnout, they would become more inclined to leave their jobs. As the PCPs have low educational levels, attractive job opportunities are rather limited [12]. Therefore, if the PCPs leave their positions in THCs, they might leave the health service industry for good, causing a huge loss of human resources. Against this backdrop, more attention should be paid to these factors to boost their satisfaction and relieve their burnout so as to reduce their turnover intention and maintain the stability of the PCP workforce [26, 38]. As an important factor affecting turnover intention, it is a priority to improve internal organizational management.

The findings also demonstrated that some personal and professional features were significant determinants of turnover intention among rural PCPs. Male PCPs were more likely to have turnover intention than female PCPs, which is possibly related to men’s strong motivation and ambition for achievement [17, 29]. Compared with formal PCPs, casual PCPs were more likely to have turnover intentions. The main explanation is that contract PCPs are only employed by THCs to relieve the healthcare staff shortage, so they have lower salaries and fewer promotion opportunities [13]. Moreover, the PCPs with 5–10 years of service showed the strongest turnover intention and this may be accounted for with their higher demands for career development [25, 38]. During this career stage, they have a strong desire to change their status and achieve success [17]. Furthermore, PCPs who worked for an average of more than 56 h per week were most likely to have turnover intention, and this finding also demonstrates that the increase of workloads after expanding the scope of public health services, and mismatch between the number of healthcare staff and workload, have led to longer working hours for the current PCPs [33, 36]. Therefore, targeted strategies should be taken to retain these PCPs.

This study was subject to a number of limitations. First, the measurement of job satisfaction, occupational burnout and turnover intention were obtained by using self-administrated questionnaires, so the self-reporting bias might have an impact on the results. Second, it was a cross-sectional study, so the interpretation of causal inference for the results was limited. It would be of benefit to confirm the causality with longitudinal data in future studies. Third, the sample was selected from three counties of one province, so the extrapolation of conclusions at the national level could be challenged.

## Conclusions

Many PCPs in rural China were dissatisfied with their job rewards, organizational management and work environment. Suffering from extremely high levels of emotional exhaustion and reduced personal accomplishment, some of them had strong intentions to quit. Job satisfaction had not only negative direct effects on burnout and turnover intention, but also an indirect effect on turnover intention through burnout as a mediator. The factors influencing turnover intention mainly included low job rewards, long working hours, heavy workloads, inferior personal value, and poor organization management within THCs. Targeting measures of improving organization management, should be taken to motivate and retain the PCPs including assigning reasonable workload, optimizing performance assessment measures and design of perfromance-based salary, encouraging decision-making participation, and establishing feedback mechanism. Such potential strategies would be beneficial to a more efficient primary care system in rural China.

## Data Availability

The data used and/or analyzed during the study are available from the corresponding author on reasonable request.

## Abbreviations

CI: Confidence intervals
CNY: Chinese Yuan
NHSR: New health system reform
ORs: Odds ratios
PCPs: Primary care providers
SEM: Structural equation modeling
THCs: Township healthcare centers
